# Comparison of Autistic Traits Between Iranian Students With Different Ethnic Backgrounds: A Cross-Cultural Study

**DOI:** 10.3389/fpsyt.2021.744180

**Published:** 2021-12-09

**Authors:** Mojtaba Elhami Athar, Ali Ebrahimi, Sirvan Karimi, Roya Esmailzadeh, Esmaeil Mousavi Asl, Morteza Azizi, Saman Heidarzadeh, Esfandiar Siahkamari, Amin Sharifi, Abbas Ramezani Farani

**Affiliations:** ^1^School of Behavioral Sciences and Mental Health (Tehran Institute of Psychiatry), Iran University of Medical Sciences, Tehran, Iran; ^2^Department of Clinical Psychology, School of Behavior Sciences, University of Social Welfare and Rehabilitation Sciences, Tehran, Iran; ^3^Department of Psychiatry, Golestan Hospital, School of Medicine, Ahvaz Jundishapur University of Medical Sciences, Ahvaz, Iran; ^4^Department of Psychology, Islamic Azad University, Sarab Branch, Sarab, Iran; ^5^Department of Psychology, Islamic Azad University, Tabriz Branch, Tabriz, Iran; ^6^Department of Psychology, Islamic Azad University, Shiraz Branch, Shiraz, Iran

**Keywords:** cultural differences, autistic traits, culture, Iran, ethnicities

## Abstract

Autistic traits (ATs) include symptoms associated with autism spectrum conditions (ASCs), which are assumed to be continuously distributed across the general population. Studies have indicated the cultural differences in the expression of ATs. Notwithstanding, our literature review indicated that studies on cross-cultural differences in the expression of ATs included samples from different countries. This is the first study designed to compare the expression of ATs between different ethnicities from the same country. Using the Autism-spectrum Quotient (AQ-28), we examined the possible cultural differences in the expression of autistic traits from four groups of students with different ethnic backgrounds, including Turkish (*n* = 262), Persian (*n* = 290), Kurdish (*n* = 300), and Luri (*n* = 307) students. Behaviors associated with autistic traits were reported overall higher for males than females. Also, significant cultural differences in autistic traits were found that were different for males and females. Furthermore, while the medical sciences student group scored significantly higher than the humanities group in the Imagination dimension, the humanities group had significantly higher scores in Number/Pattern dimensions than the engineering and medical sciences groups. Altogether, our results provide further support for the idea that the expression of ATs is significantly influenced by culture. A significant limitation of the current study was that groups were not matched with respect to age, percentage of male participants, and fields of studies and that these variables may influence the AQ scores.

## Introduction

Autistic traits (ATs) include symptoms associated with autism spectrum conditions (ASCs), which are assumed to be continuously distributed in the general population. This extension into the general population suggests a dimensional approach and presence of a broad autism phenotype—a continuum ranging from those with no autistic traits to those facing significant challenges in their everyday lives (1, 2). While core autistic traits are believed to be universal, evidence suggests that there are cultural differences in the expression, identification, and/or reporting of symptomatology (3–6). Unique cultural stigmas, norms, and preferences may conceal or highlight relative differences between autistic traits and typically developing behaviors (3, 7).

In recent years, many studies have been conducted in various countries and cultures to examine the cultural differences in the expression of ATs (4–6, 8–21). Taken together, the results of these studies indicate that the expression of autistic traits is influenced by the ethnicity and cultural context of individuals. For instance, Freeth et al. (3) administered the Autism-spectrum Quotient (AQ) on a sample of neurotypical individuals from one Western culture (UK) and two Eastern cultures (India and Malaysia) to examine cultural differences in the expression of ATs. Their results indicated that behaviors associated with autistic traits were significantly higher in Eastern cultures than the Western culture. Similarly, Carruthers et al. (4) indicated that different sets of AQ items demonstrated excellent discriminatory power in the Indian, Japanese, and UK samples for predicting an autism diagnosis, highlighting the cultural differences in certain autistic traits.

In addition, it has been indicated that the expression of ATs is influenced by gender differences. For instance, Baron-Cohen et al. (22) reported that males had higher Autism-Spectrum Quotient (AQ) total score means than females and scored higher on all AQ subscales, including social skills, attention switching, attention-to-detail, communication, and imagination, with noticeable elevation on the attention-to-detail subscale. These findings make a case for the speculation that altogether males have higher AT levels than females in the general population. Several subsequent studies supported these findings (2, 5, 6, 23–25), though some failed to replicate such findings (12, 26).

Further, Baron-Cohen et al. (22) found that ATs differ significantly with respect to individuals' fields of study. Accordingly, physics, engineering, and mathematics students had more ATs than students in the humanities and social sciences. In line with the hypothesis that autistic traits may be associated with scientific skills (27), it is assumed that in contrast to individuals in science, both the humanities and social sciences disciplines emphasize human interaction and are likely to be appealing for students who like social engagement and a high-level concentration on topics such as economics and political science. These findings supported the idea that autistic traits are associated with areas of study (15).

### Iranian Context

Iran is a country with various ethnic and linguistic groups, each with its own indigenous language and unique traditions or folklores unified through a shared Iranian nationality. The majority of the population speaks Persian, which is also the official language of the country. Besides, in northern Iran, mostly confined to Gilan and Mazandaran, the Gilaki and Mazandarani languages are widely spoken. Turkish comprise the largest minority ethnic group in Iran, extending from north to south of Iran, where the Turkish language is widely spoken. In addition, varieties of Kurdish are widely spoken in the province of Kurdistan and nearby areas. In Khuzestan, several distinct varieties of Persian are spoken, and Luri and Lari are also spoken in southern Iran. Also, Iran is a country with various religions. For instance, Twelver Shia Islam is the official state religion to which most of the population adhere. Other minorities include Sunni Muslims, mainly Kurds and Baloches, and non-Muslim religious minorities, including Christians, Zoroastrians, Jews, Bahá'ís, Mandeans, and Yarsanis. Therefore, it is highly probable that different cultural values and norms among ethnicities in Iran may cover or highlight differences between those with different autistic trait levels.

Our literature review indicated that studies on cross-cultural differences in the expression of ATs included samples from different countries. The current study is informative in that it contains samples of different ethnicities from the same country. This study aims to examine autistic traits among four ethnicities in Iran, including Turkish, Persian, Kurdish, and Luri ethnicities. Our first goal in this study is to examine the differences in the distribution of ATs among the four ethnicities. Second, we will explore gender differences in autistic traits for each ethnic group separately. Finally, the present study will examine whether there are differences in ATs based on the field of study.

## Materials and Methods

### Participants

Participants were 1,159 (aged 18–60, *M*: 26.40, *SD*: 7.74; 44.1% men) university students from Turkish (*n* = 262), Persian (*n* = 290), Kurdish (*n* = 303), and Luri (*n* = 307) ethnicities who were recruited in 2021 (see Table 1 for more information on demographic characteristics). Questionnaires were distributed to 1,200 students, and 1,162 completed questionnaires were received (response rate: 96.58%). The participants were compared with respect to demographic information. As shown in Table 1, the result of the Chi-Square test indicated that the groups were matched with respect to total sample size [*X*^2^ = (3, *n* = 1,162) = 4.272, *p* = 0.23], the percentage of females in each ethnicity group [*X*^2^ = (3, *n* = 646) = 7.251, *p* = 0.064], and the percentage of participants in the science field of study [*X*^2^ = (3, *n* = 205) = 5.127, *p* = 0.163], while they were not matched with respect to the percentage of male participants [*X*^2^ = (3, *n* = 516) = 23.364, *p* = 0.001], humanities/social science [*X*^2^ = (3, *n* = 506) = 15.945, *p* = 0.001], and medicine [*X*^2^ = (3, *n* = 506) = 7.251, *p* = 0.001] fields of study. *Post-hoc* tests, using standardized residuals, indicated that the percentage of male participants was significantly higher in the Luri group than the Persian and Turkish groups (*p* < 0.01), while a significant difference was not observed between the Luri group and the Kurdish group (*p* > 0.05), and between the Kurdish and Persian and Turkish groups (*p* > 0.05). Similarly, the percentage of participants in the Humanities/Social science field was significantly higher in the Persian group than in the other three ethnic groups (*p* < 0.05), and it was significantly lower in the Luri group compared to the rest of the ethnic groups (*p* < 0.05). In the same vein, the percentage of participants in the Medicine field of study was significantly higher in the Luri group compared to the other three groups (*p* < 0.05) and was significantly lower in the Persian groups compared to the rest of the ethnic groups (*p* < 0.05). Also, the results of the ANOVA test indicated that the groups were not matched with respect to the age variable [F_(3, 1151)_ = 26.29, *p* = 0.001, $\eta_{\text{p}}^{2}$ = 0.064]. The subsequent *Post-hoc* Tukey test results with a Bonferroni adjusted alpha level of 0.0125 per test (0.05/4) indicated that the Luri group (*M*: 29.42, *SD*: 8.15) was significantly older than Persian (*M*: 24.67, *SD*: 7.14), Turkish (*M*: 24.62, *SD*: 6.78), and Kurdish (*M*: 26.47, *SD*: 7.88) groups. Further, the Kurdish group had a significantly higher age than the Turkish and Persian groups, while there was no significant difference with respect to the age variable between the Turkish and Persian groups.

**Table 1:** Demographic information.

**Variables**	**Ethnicities**				
	**Turkish**	**Persian**	**Kurdish**	**Luri**	***P***
*N*	262	290	303	307	0.234
**Gender**	1 missing	–	–	2 missing	
Male	102 (38.9%)	105 (36.2%)	138 (44.4%)	168 (55%)	0.001
Female	159 (61.1%)	185 (63.8%)	165 (55.6%)	137 (45%)	0.064
**Age Mean (** ***SD*** **)**	24.62 (6.78)	24.67 (7.14)	26.47 (7.88)	29.42 (8.14)	0.001
**Area of study**	4 missing	10 missing	4 missing	4 missing	
Humanities/Social science	109 (42.1%)	153 (54.6%)	144 (48.16%)	100 (21.5%)	0.001
Medicine	101 (39.4%)	79 (28.2%)	111 (37.12%)	138 (45.5%)	0.001
Science	48 (18.5%)	48 (17.1%)	44 (14.71%)	65 (21.5%)	0.163

*M, Mean; SD, Standard Deviation; AQ, Autism-spectrum Quotient*.

### Procedure

At first, the ethics committee of the Iran University of Medical Sciences approved this study (code number: IR.IUMS.REC 1395.95-04-185-29338). Then, a demographic form with four questions assessing age, gender, ethnicity (i.e., Turkish, Persian, Kurdish, Luri), and field of study (Humanities/Social science, Medicine, Science) and a twenty-eight-item Likert online survey [AQ-28; (28)] were developed and administered using Google Forms. The researchers shared the online questionnaires in the social media groups of universities. Participants provided online informed consent after reading the study purpose and being assured about confidentiality issues. Then they were asked to complete the questionnaires. Inclusion criteria included studying at undergraduate or graduate levels, the age range of > 18, and being interested in participating in the study.

### Measure

#### The Short-Form of the Autistic Spectrum Quotient Questionnaire (AQ-28)

The short form of the autistic spectrum quotient questionnaire [AQ-28; (28)] consisted of 28 statements about personal preferences and habits in the five areas reflecting the autism phenotype, including social skills, routine, switching, imagination, and numbers/patterns. Participants complete the questionnaire on a 4-point Likert scale. AQ-28 score ranges from 28 to 112. Ebrahimi et al. (29) supported the two higher-order factors models, including “social behavior” and numbers/patterns for the Persian AQ-28. Furthermore, the internal consistency of Persian AQ-28 subscales scores ranged from 0.40 (Routine) to 0.78 (Social Behavior).

### Data Analysis

In the present study, for data entry and statistical analyses, SPSS 20 software was used. Descriptive information for all variables used in the present study is presented in Table 1. Data were analyzed using Independent *T*-test and Univariate Variance Analysis (ANOVA). A series of ANOVAs and *post-hoc t*-tests were performed to examine whether there were significant differences in AQ scores between cultural groups (separately for males and females), gender groups, and areas of studies. Also, independent *T*-tests were performed to examine the intra ethnic gender differences in AQ scores for each ethnicity. A *p* level of < 0.05 was considered as indicating statistically significant results.

## Results

### The Effect of Gender on AQ Scores

A 2 × 4 (gender × ethnicity) between subjects ANOVA found a main effect of gender on Routine (areas reflecting the autism phenotype) *F*_(1, 1, 138)_ = 8.08, *p* = 0.001, $\eta_{\text{p}}^{2}$ = 0.007 with males (*M* = 2.64, *SD*: 1.00) scoring higher than females (mea*n* = 2.61, *SD*: 1.06) overall. Similarly, there was a significant main effect of gender on Imagination (another indicator of AT) [*F*_(1, 1, 123)_ = 10.22, *p* = 0.001, $\eta_{\text{p}}^{2}$ = 0.009], though females (*M* = 5.76, *SD*: 1.72) had higher scores than males (*M* = 5.57, *SD*: 1.68) totally. Finally, the results yielded a main effect of gender on the Number/Pattern dimension, such that males (*M* = 2.39, *SD*: 1.43) scored higher than females (*M* = 2.25, *SD*: 1.40) overall. There was no significant gender × ethnicity interaction on AQ Total and dimensions scores.

### Intra Ethnic Gender Differences in AQ Scores

**Figure 1:**
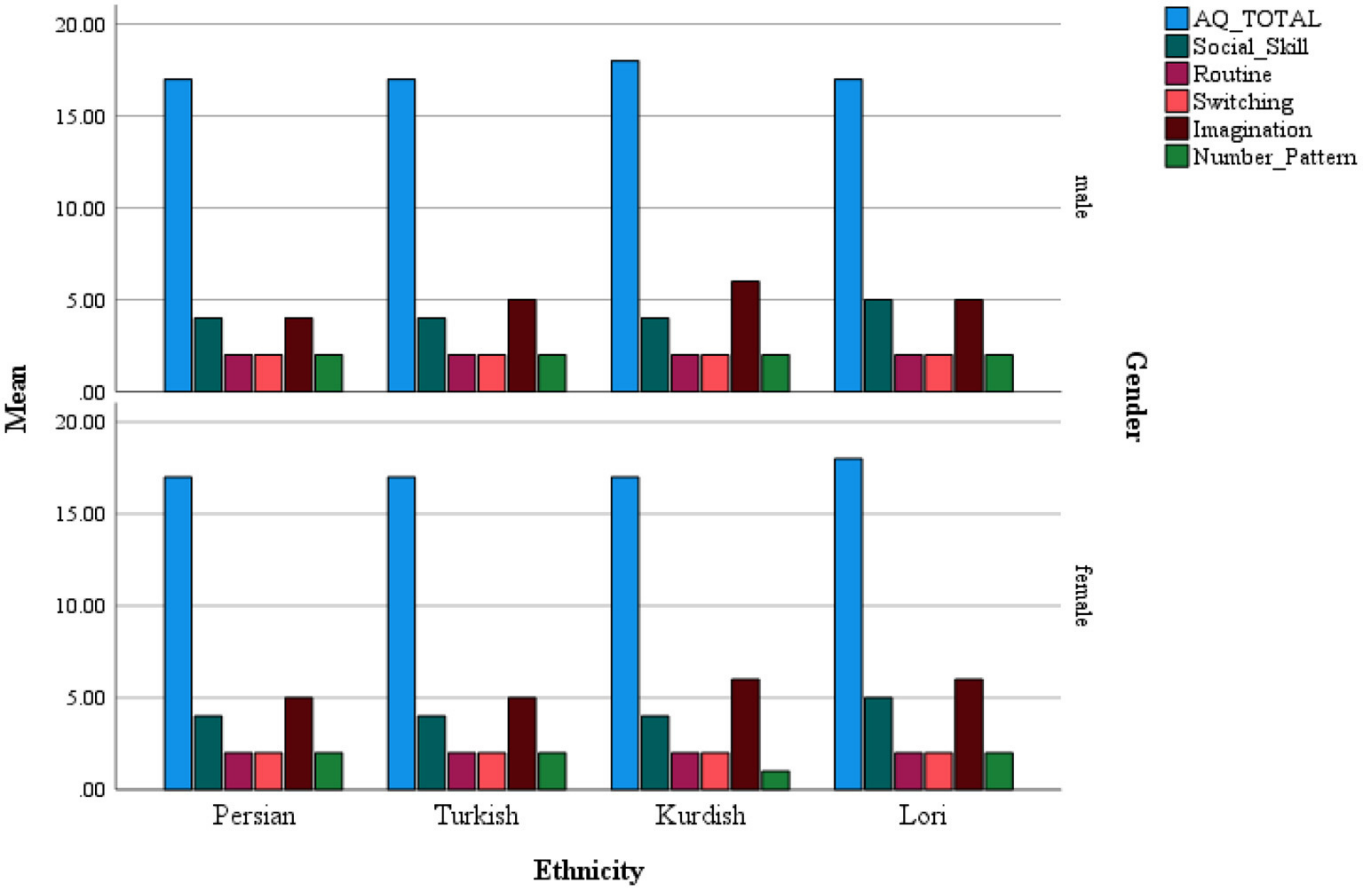
Distribution of AQ scores across ethnicities for males and females.

A series of independent *T*-tests were conducted to examine the intra ethnic gender differences in AQ scores for each ethnicity. The results indicated that in the Persian group, males (*M*: 2.96, *SD*: 1.32) scored significantly higher [*t*_(225)_: 2.67, *p* < 0.05, *d*:0.25] in the Number/Pattern dimension than females (*M*: 2.63, *SD*: 1.41). For the Turkish group, the results indicated a statistically significant difference in Routine [*t*_(254)_: 2.98, *p* < 0.05, *d*: 0.38) and Imagination [*t*_(246)_: −2.25, *p* < 0.05, *d*: −0.32) dimensions for males and females, that is, males (*M*: 2.55, *SD*: 0.96) scored significantly higher than females (*M*: 2.11, *SD*: 0.96) in the Routine dimension; conversely, in the Imagination subscale, females (*M*: 5.67, *SD*: 1.76) scored significantly higher than males (*M*: 5.08, *SD*: 1.78). With respect to the Kurdish group, the results of the independent *t*-test showed that males scored significantly higher than females in Switching, *t*_(352)_: 1.99, *p* < 0.05, *d*: 0.21 [*M*: 2.66, *SD*: 0.94/(*M*: 2.46, *SD*: 1.10)], Number/Pattern, *t*_(355)_: 1.97, *p* < 0.05, *d*: 0.21 [*M*: 2.41, *SD*: 1.35/(*M*: 2.11, *S.D*: 1.38)], and AQ Total score, *t*_(337)_: 1.99, *p* < 0.05, *d*: 0.22 [*M*: 18.42, *SD*: 3.64)/(*M*: 17.59, *SD*: 3.96)]. Finally, in the Luri culture, males (*M*: 2.52, *SD*: 0.93) scored significantly higher in the Routine dimension [*t*_(303)_: 2.00, *p* < 0.05, *d*: 0.23] than females (*M*: 2.29, *SD*: 1.03); however, females (*M*: 6.27, *SD*: 1.58) had significantly higher scores in the Imagination dimension [*t*_(300)_: −2.36, *p* < 0.05, *d*: −0.27) than males (*M*: 5.81, *SD*: 1.63) (Figure 1). Given that there were significant intra ethnic gender differences in AQ scores, we decided to examine the cultural difference in AQ scores separately for both genders.

### Cultural Differences in AQ Scores Between Males and Females

While there was no interaction between ethnicity and gender, we decided to conduct separate analyses for males and females as exploratory analyses. Results of one-way ANOVA indicated that scores of Social Skill [*F*_(3, 504)_ = 5.87, *p* < 0.001, $\eta_{\text{p}}^{2}$ = 0.03], Imagination (*F*_(3, 499)_ = 15.25, *p* < 0.001, $\eta_{\text{p}}^{2}$ = 0.08], and Number/Pattern [*F*_(3, 500)_ = 11.07, *p* < 0.001, $\eta_{\text{p}}^{2}$ = 0.06] dimensions differed between the ethnicities for males. *Post-hoc* Tukey tests with a Bonferroni adjusted alpha level of 0.0125 per test (0.05/4) indicated that the Social Skill dimension score was significantly higher for Luri students (*M*: 5.03, *SD*: 1.59) than Persian (*M*: 4.36, *SD*: 1.37) and Turkish (*M:* 4.31, *SD:* 1.54) students. In addition, Kurdish (*M*: 6.09, *SD*: 1.56) and Luri (*M*: 5.81, *SD*: 1.63) students had significantly higher Imagination scores than Persian (*M*: 4.96, *SD*: 1.58) and Turkish (*M*: 5.08, *SD*: 1.76) students. With regard to the Number/Pattern dimension, Persian (*M*: 2.96, *SD*: 1.32) and Turkish (*M*: 2.42, *SD*: 1.31) students scored significantly higher than Luri students (*M*: 1.99, *SD*: 1.51); however, only Persian students had significantly higher Number/Pattern scores than Kurdish (*M*: 2.41, *SD*: 1.35) students.

Likewise, a one-way ANOVA was conducted to examine the cultural differences in AQ scores for females. Results indicated that there are significant differences between groups in Social Skill [*F*_(3, 632)_ = 5.71, *p* < 0.001, $\eta_{\text{p}}^{2}$ = 0.03), Routine [*F*_(3, 633)_ = 4.68, *p* < 0.001, $\eta_{\text{p}}^{2}$ = 0.02], Imagination [*F*_(3, 626)_ = 14.42, *p* < 0.001, $\eta_{\text{p}}^{2}$ = 0.06], and Number/Pattern [*F*_(3, 633)_ = 7.55, *p* < 0.001] dimensions scores. The subsequent *Post-hoc* Tukey test results with a Bonferroni adjusted alpha level of 0.0125 per test (0.05/4) indicated that the Persian group (*M*: 4.36, *SD*: 1.62) had a significantly lower Social Skills score than Kurdish (*M*: 4.79, *SD*: 1.58) and Luri (*M*: 5.01, *SD*: 1.71) students. Conversely, Persian students (*M*: 2.54, *SD*: 1.05) scored higher in AQ Routine dimension than Turkish (*M*: 2.11, *SD*: 0.97) and Kurdish (*M*: 2.23, *SD*: 0.99) students. With regard to the Imagination subscale, Luri (*M*: 6.27, *SD*: 1.58) and Kurdish (*M*: 5.96, *SD*: 1.61) groups had significantly higher scores than Turkish (*M*: 5.56, *SD*: 1.79) and Persian (*M*: 5.25, *SD*: 1.76) groups, while there were no significant differences between the latter two groups. Finally, the results indicated that the Persian group (*M*: 2.63, *SD*: 1.41) had a significantly higher Number/Pattern scores than Turkish (*M*: 2.18, *SD*: 1.38), Kurdish (*M*: 1.98, *SD*: 1.33), and Luri (*M*: 2.05, *SD*: 1.38) groups (Table 1).

### Effect of Areas of Study Differences on AQ Scores

**Figure 2:**
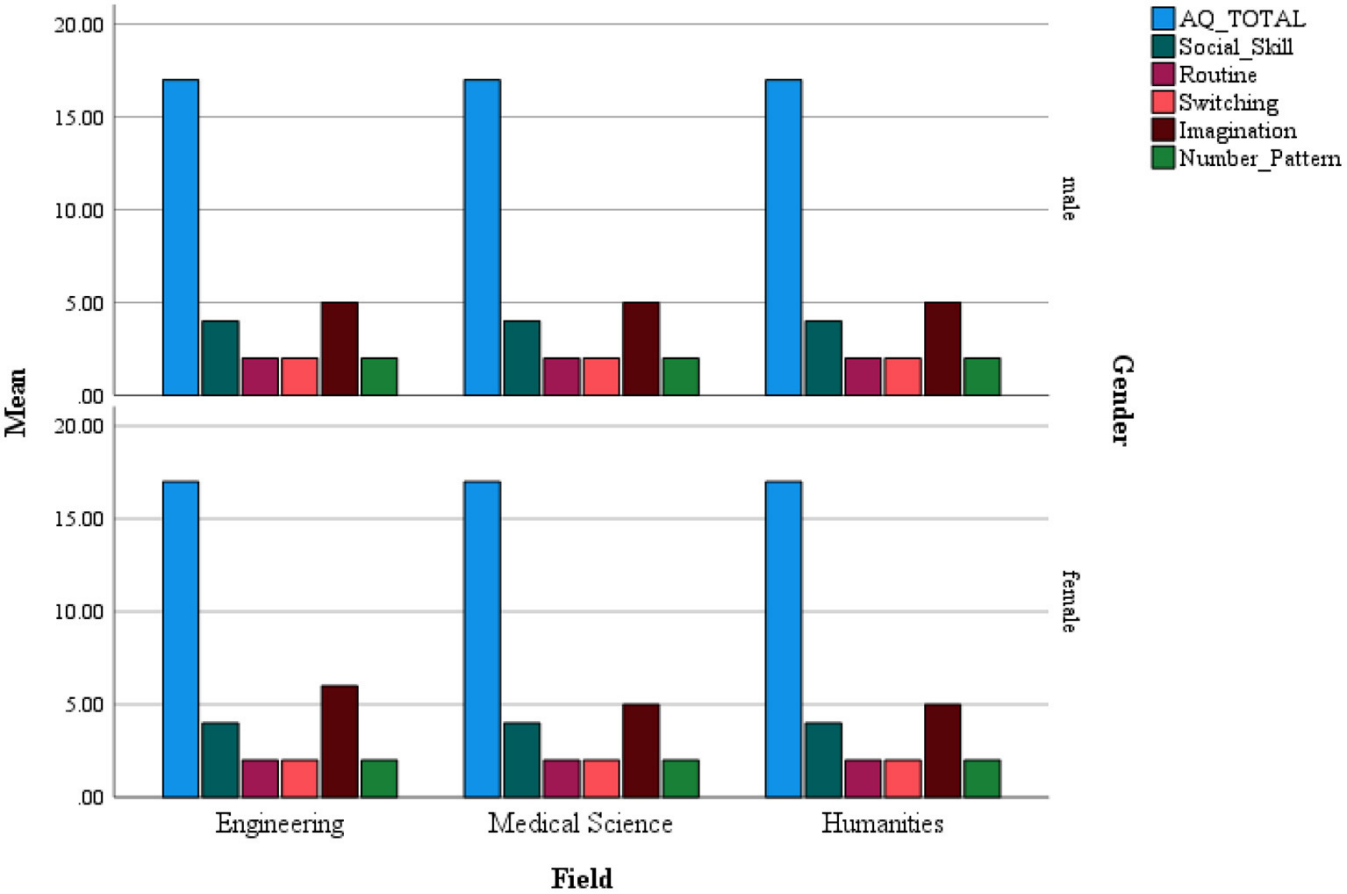
Distribution of AQ scores across fields of study for males and females.

Results of one-way ANOVA indicated significant differences across areas of study in scores of Social Skill [*F*_(2, 1, 120)_ = 3.26, *p* < 0.05, $\eta_{\text{p}}^{2}$ = 0.006], Imagination [*F*_(2, 1, 110)_ = 3.26, *p* < 0.05, $\eta_{\text{p}}^{2}$ = 0.008], and the Number/Pattern dimensions [*F*_(2, 1, 118)_ = 8.66, *p* < 0.001, $\eta_{\text{p}}^{2}$ = 0.01]. *Post-hoc* Tukey test with a Bonferroni adjusted alpha level of 0.016 per test (0.05/3) showed that there were no significant differences in the Social Skill dimension between the groups. On the other hand, in the Imagination dimension, Medical Sciences students (*M*: 5.84, *SD*: 1.68) scored significantly higher than Humanities (*M*: 5.55, *SD*: 1.71) group. Also, in the Number/Pattern dimensions, the Humanities group (*M*: 2.50, *SD*: 1.34) had a significantly higher score than Engineering (*M*: 2.14, *SD*: 1.43) and Medical Sciences (*M*: 2.15, *SD*: 1.46) groups (Figure 2).

## Discussion

The current study aimed to compare Autistic Traits between Iranian university students with different ethnical backgrounds. We studied the ATs differences based on gender for each ethnicity separately; differences in ATs based on study fields were examined as well. Available data indicates cross-cultural differences in the expression of ATs (3, 4, 6, 8, 9, 12–14, 17, 18, 20). Similarly, gender differences (2, 5, 6, 23–25) and the variations in ATs based on the field of study (14, 22) have been supported in previous studies. Our results are discussed in regard to ATs differences according to gender, ethnicity, and the field of study, followed by a reflection upon the findings of previous studies.

### Gender Differences in ATs

Our results indicated gender differences in Routine, Imagination, and Number/Pattern dimensions scores. While males had higher Routine and Number/Pattern scores, they scored lower than females in Imagination. Our analysis brought some interesting results with respect to sex differences in ATs across ethnicities. Consistent with previous studies (2, 5, 6, 23–25), we found predominant autistic traits for males across different ethnicities. In the Persian group, males scored significantly higher in the Number/Pattern dimension than females. Similarly, Turkish males scored significantly higher than females in the Routine dimension. With respect to the Kurdish group, males scored significantly higher than females in Switching, Number/Pattern, and AQ Total score. Finally, in the Luri culture, males scored significantly higher in the Routine dimension. These results are consistent with the finding that AT levels are lower in females than males (3, 30). However, contrary to our expectation, Turkish and Luri females scored higher in the Imagination dimension than males. Notwithstanding, not all researchers have confirmed sex differences in AQ scores (11, 31). For instance, in Hurst et al. (11) study, females scored slightly higher than males in the AQ Total score and most subscales, though the differences were not statistically significant. All in all, our results suggest that while males had overall higher AT levelsthan females, gender differences in ATs were not in the same pattern across Iranian ethnicities. Also, contrary to our hypothesis, Turkish and Luri females scored higher in the Imagination dimension than males. Therefore, future studies are recommended to examine the underlying cultural differences that are likely to result in these incongruences.

### Cultural Differences in AQ Scores

With respect to cultural differences in AQ scores, which we analyzed separately for both genders, the results indicated significant cultural differences in AQ dimensions. Concerning male groups, Luri students had significantly higher Social Skills dimension scores than Persian and Turkish students, while the mean Number/Pattern dimension score was significantly lower for the Luri group than Persian and Turkish students. In addition, Kurdish and Luri students had significantly higher Imagination scores than Persian and Turkish students. Only Persian students had significantly higher Number/Pattern scores than Kurdish students. With respect to the female sample, the Persian group had a significantly lower Social Skills score than Kurdish and Luri students but scored higher in AQ Routine dimension than Turkish and Kurdish students. In the Imagination subscale, Luri and Kurdish groups had significantly higher scores than Turkish and Persian groups. Finally, for the Number/Pattern subscale, our results indicated that the Persian group had a significantly higher score than the Turkish, Kurdish, and Luri groups. These results are consistent with previous studies on the cross-cultural differences in ATs. For instance, Freeth et al. (3) indicated that behaviors associated with ATs were reported significantly higher in the Eastern cultures (India and Malaysia) than the Western culture (UK). Similarly, Carruthers et al. (4) administered the AQ among Indian, Japanese, and UK samples and found that that different sets of AQ items had excellent discriminatory power in predicting an autism diagnosis in each sample, which accentuates the cultural differences in certain autistic traits. Unfortunately, since no study has examined the cultural differences of psychological constructs among Iranian ethnicities, our ability to discuss the results is too limited. Notwithstanding, the influence of socioeconomic status on the expression of ATs has been reported in previous studies (32, 33). For instance, Suzuki et al. (32) indicated that in Japan, except for the numbers/patterns dimension, individuals with lower socioeconomic status had significantly higher AQ total and dimension scores than their respective counterparts (i.e., those with high socioeconomic status). While we did not measure the participants' SES in the current study, previous studies indicated significant SES differences among Iranian ethnicities. For instance, Tehran (where most of the Persians live) has resulted in a wide socioeconomic gap between the center and the peripheries (e.g., Kurdistan and Lorestan) due to a highly centralized development strategy (34). In addition, Turkish resident cities such as Tabriz and Urmia have been developed to a high SES rank in the recent decade. In this concern, our results align with Suzuki et al. (32) in that the participants from low SES ethnicities (in our study Kurdish and Luri participants) had overall higher AQ scores, while the Persian group that enjoys high SES had significantly higher Number/Pattern scores than the rest of the groups.

### Effect of Areas of Study Differences on AQ Scores

Our results indicated that while there were no significant differences in the Social Skill dimension between the groups, Medical Sciences students scored significantly more than the Humanities group in the Imagination dimension. On the other hand, the Humanity group had significantly high scores in Number/Pattern dimensions than Engineering and Medical Sciences groups. Our result failed to replicate the previously proposed finding that students in Science majors (e.g., engineering) have significantly higher ATs than non-science students (3, 14, 22). However, in line with our results, Pisula et al. (12) found that humanities students had higher ATs than social sciences and/or medical students. They hypothesized that the results were influenced by the large presence of students of classical studies and applied linguistics among the humanities students who might demonstrate some traits common for autism conditions, e.g., the tendency for in-depth, detailed analysis of the material (in their case text). The propensity for systemizing typical of autism conditions (35) can also be pronounced in some students of applied linguistics, who may be interested in language as a system.

Our results should be interpreted considering the following limitations. First, besides the ethnicities examined in the current study, there are other ethnicities (e.g., Arabs, Baloch) that we did not study because of the lack of access. Future studies are recommended to include and study ATs in these ethnicities as well. Second, some ethnic groups were not matched on some demographic variables (e.g., gender and field of study) that may affect the AQ results and interpretation. Third, for data gathering, we used only the self-report method; using other assessment methods is recommended for future studies. Fourth, our study included only a student sample, and we did not examine cross-cultural differences of ATs among the community and clinical samples from different ethnicities, so future studies are recommended to examine and compare ATs across ethnicities with community or clinical samples. Finally, we did not measure the socioeconomic status (SES) of the participants, which has been shown to influence the expression of ATs (32).

## Conclusion

To our knowledge, the current study is the first to examine the cultural differences in ATs within various cultures in the same country. Our results indicated that the expression of autistic traits is influenced by culture and that while there are gender differences in ATs across Iranian ethnicities (higher AT levelsfor males), the pattern of differences was not the same among the ethnicities. Also, contrary to our hypothesis, our results did not support the idea that students of the science field have more ATs than humanities and social sciences. The current study had a couple of strengths. First, our study included a large study sample size consisting of different Iranian ethnicities. Second, we examined the cross-cultural differences in ATs separately for males and females, which provided fruitful information, indicating that the cross-cultural differences in ATs are not the same for males and females.

## Data Availability Statement

The raw data supporting the conclusions of this article will be made available by the authors, without undue reservation.

## Ethics Statement

The studies involving human participants were reviewed and approved by School of Behavioral Sciences and Mental Health (Tehran Institute Psychiatry), Iran University of Medical Sciences, Tehran, Iran. The patients/participants provided their written informed consent to participate in this study.

## Author Contributions

ME and AE: prepared the manuscript and performed the data analysis. SK, RE, EM, MA, SH, ES, AS, and AR: gathered data. All authors contributed to the article and approved the submitted version.

## Funding

This study was financially supported by the School of Behavioral Sciences and Mental Health (Tehran Institute Psychiatry), Iran University of Medical Sciences, Tehran, Iran (grant number 3367).

## Conflict of Interest

The authors declare that the research was conducted in the absence of any commercial or financial relationships that could be construed as a potential conflict of interest.

## Publisher's Note

All claims expressed in this article are solely those of the authors and do not necessarily represent those of their affiliated organizations, or those of the publisher, the editors and the reviewers. Any product that may be evaluated in this article, or claim that may be made by its manufacturer, is not guaranteed or endorsed by the publisher.
